# TinyML-Sensor for Shelf Life Estimation of Fresh Date Fruits

**DOI:** 10.3390/s23167081

**Published:** 2023-08-10

**Authors:** Ramasamy Srinivasagan, Maged Mohammed, Ali Alzahrani

**Affiliations:** 1 Department of Computer Engineering, College of Computer Sciences and Information Technology, King Faisal University, Al Hofuf 36362, Saudi Arabia; aalzahrani@kfu.edu.sa; 2 Date Palm Research Center of Excellence, King Faisal University, Al Hofuf 36362, Saudi Arabia; memohammed@kfu.edu.sa; 3 Agricultural and Biosystems Engineering Department, Faculty of Agriculture, Menoufia University, Shebin El Koum 32514, Egypt

**Keywords:** artificial intelligence (AI), TinyML, edge computing, modified atmosphere, prediction models, Short-Wave Near-Infrared, food supply chain, regression models

## Abstract

Fresh dates have a limited shelf life and are susceptible to spoilage, which can lead to economic losses for producers and suppliers. The problem of accurate shelf life estimation for fresh dates is essential for various stakeholders involved in the production, supply, and consumption of dates. Modified atmosphere packaging (MAP) is one of the essential methods that improves the quality and increases the shelf life of fresh dates by reducing the rate of ripening. Therefore, this study aims to apply fast and cost-effective non-destructive techniques based on machine learning (ML) to predict and estimate the shelf life of stored fresh date fruits under different conditions. Predicting and estimating the shelf life of stored date fruits is essential for scheduling them for consumption at the right time in the supply chain to benefit from the nutritional advantages of fresh dates. The study observed the physicochemical attributes of fresh date fruits, including moisture content, total soluble solids, sugar content, tannin content, pH, and firmness, during storage in a vacuum and MAP at 5 and 24 °C every 7 days to determine the shelf life using a non-destructive approach. TinyML-compatible regression models were employed to predict the stages of fruit development during the storage period. The decrease in the shelf life of the fruits begins when they transition from the Khalal stage to the Rutab stage, and the shelf life ends when they start to spoil or ripen to the Tamr stage. Low-cost Visible–Near–Infrared (VisNIR) spectral sensors (AS7265x—multi-spectral) were used to capture the internal physicochemical attributes of the fresh fruit. Regression models were employed for shelf life estimation. The findings indicated that vacuum and modified atmosphere packaging with 20% CO_2_ and N balance efficiently increased the shelf life of the stored fresh fruit to 53 days and 44 days, respectively, when maintained at 5 °C. However, the shelf life decreased to 44 and 23 days when the vacuum and modified atmosphere packaging with 20% CO_2_ and N balance were maintained at room temperature (24 °C). Edge Impulse supports the training and deployment of models on low-cost microcontrollers, which can be used to predict real-time estimations of the shelf life of fresh dates using TinyML sensors.

## 1. Introduction

Substantial losses occur within the food product supply chain, especially in fruits and vegetables, from when they are cultivated until they reach the consumer. A considerable quantity of food is produced but is not consumed due to these losses in the supply chain. This segment of postharvest losses is approximately 25–30% [1]. Roughly 14 percent of the world’s food is lost between harvest and retail, and another 17 percent is wasted in retail and at the consumption level. The 2030 Agenda for Sustainable Development, specifically SDG 12, Target 12.3, aims to halve per capita global food waste at the retail and consumer levels and reduce food losses along production and supply chains [1].

The date palm (*Phoenix dactylifera* L.) is one of the oldest fruit trees that grows widely in the Middle East and North Africa. Dates are a key source of income and a staple food for locals in many regions where they are cultivated. They have also played an essential role in those countries’ socioeconomic and environmental conditions [2]. Consequently, the demand for date-importing countries like India, Germany, the United Kingdom, the USA, the Netherlands, Canada, Spain, Italy, Belgium, and Switzerland has increased significantly. Given the importance of the date palm trade on a local and global scale, ensuring a continuous supply in the market is vital [3].

A total of 8.46 million tons of date fruit are produced annually worldwide. In 2022, Saudi Arabia exported 1.54 million tonnes. Date fruit is renowned for its high nutritional value, and clinical studies have proven that consuming two to three servings of date fruit per day is beneficial for patients with Type-2 diabetes due to its low Glycemic Index (GI). Consuming foods with a GI index of less than 50 is recommended. Date varieties such as Khalas cv., Hilali, Sukkary, Sagai, and Shaqra have a GI of less than 50 and are considered safe to consume when they reach the mature stage. Fresh dates are suggested as a healthy and nutritious snack because they have a lower calorie and sugar content compared with dried dates (Tamr). The GI of fresh fruits is 2–3 times lower than that of mature dates, and they are also rich in calcium and other nutrients. However, all varieties of fresh dates can be consumed without restrictions (regarding the number of servings per day that may increase Glycemic levels) if consumed before reaching the mature stage. It is important to note that the shelf life of fresh fruits is only 10–12 days before reaching maturity in an uncontrolled environment [4,5,6].

Dates fruit is known for its great nutritional value, and it has been proven in a clinical study that two to three servings of dates fruit per day are beneficial for patients with diabetes (Type-2) because of their low Glycemic Index (GI). A GI index of less than 50 is recommended for consumption [4]. The dates varieties, Khalas cv., Hilali, Sukkary, Sagai, and Shaqra, have a GI of less than 50 and are very safe to consume at the mature stage. Fresh dates are recommended as a healthy and nutritious snack due to their low calorie and sugar content compared with dried dates (Tamr). The GI of fresh fruits is 2–3 order times less than that of mature fruits and is rich in calcium apart from its nutritional content. However, if consumed before the mature stage, all the fresh date varieties can be consumed without restrictions (number of servings in a day, which will not increase GL-Glycemic Level).

Nonetheless, fresh date fruits exhibit seasonality and are available from July to November. Consequently, storing these fruits under appropriate conditions is imperative to ensure food security. The key objectives of fruit storage encompass preserving fruits for consumption beyond their regular season, maintaining the quality of the food, slowing down the decaying process, ensuring a steady supply to the market, and obtaining better pricing [7,8,9].

The dates’ freshness and shelf life depend on preserving their physicochemical properties, including moisture content (MC), total soluble solids (TSS), firmness, pH, and water activity (AW). However, the conventional methods of analyzing these properties and other quality indicators in the fruit are time-consuming, labor-intensive, and damaging. Consequently, the need for rapid and non-destructive testing of fruit quality has emerged as a crucial area of research. Non-destructive techniques, such as spectroscopic and imaging methods, have proven highly effective in food control. These analytical techniques offer numerous advantages, including preserving samples, producing swift results, and conducting checks during production processes. As a result, they have been extensively studied and utilized in the agro-food sector for a considerable period of time [10].

The most commonly widespread non-destructive techniques in the food industry are indeed visual/Near-Infrared (NIR) spectroscopy, NIR spectroscopy (NIRs), and image and multi/hyperspectral analysis [11]. The hyperspectral imaging technique to detect fungal contamination of edible date fruits using Latent Discriminant Analysis and Quadratic Discriminant Analysis was investigated in [12]. This work evaluates the possibility of an objective, fast, and non-destructive method to identify healthy and fungal-infected date fruits. Four wavelengths (1120, 1300, 1610, and 1650 nm) were used for this study [12].

Visible (Vis; 400–750 nm) and near-infrared red (NIR; 750–2500 nm) region spectroscopy have been employed to evaluate the quality and internal attributes of fruits and vegetables. Focusing specifically on “point” spectroscopy rather than hyperspectral imaging, various non-destructive testing applications have been successfully concluded using this approach [13]. Several researchers have used spectral bands (Visible, Short-Wave Near-Infrared (SWNIR), NIR, and IR) to observe the reflectance properties to estimate the quality or shelf life of various fruits such as Kesar mango [14], grapes [15], persimmon [16], muskmelon [17], kiwi fruit [18], strawberry [19], Royal Gala [20], and pineapple [21].

The researchers [17,22,23,24,25,26,27,28,29] have put forth a collection of deep learning (DL) and machine learning (ML) techniques to classify and grade the quality of date fruits. However, these approaches predominantly rely on cloud services for training and inference, making them unsuitable for edge computing. In contrast, edge artificial intelligence (AI) primarily processes data locally, reducing internet data transfer and conserving significant bandwidth. Moreover, edge computing devices are designed for highly efficient power consumption, resulting in lower power requirements than cloud data centers. To meet the demands of edge AI computing, we propose adopting lightweight AI models that can be deployed on microcontrollers, a new paradigm of Edge AI computing referred to as Tiny Machine Learning (TinyML). This approach satisfies all the requirements for edge AI computing and enables efficient processing and classification of date fruit quality without relying on resource-intensive cloud services [30,31].

Understanding the shelf life of perishable food products is essential for ensuring food safety and quality and reducing food waste. Fresh dates have a limited shelf life and are susceptible to spoilage, which can lead to economic losses for producers and suppliers. The problem of accurate shelf life estimation for fresh dates is essential for various stakeholders involved in the production, supply, and consumption of dates [32,33]. By addressing this problem, we can enhance food safety, reduce waste, and improve the overall efficiency and sustainability of the food industry. By addressing this problem and developing a reliable methodology for estimating the shelf life of fresh dates, we aim to provide a practical solution to enhance the quality control processes throughout the supply chain. This can benefit producers, suppliers, and consumers by reducing food waste, improving product quality, and ensuring that dates reach consumers at their peak freshness. Accurately estimating shelf life also has broader implications for the food industry. It can enable better inventory management, reduce losses, and optimize production and distribution processes. Additionally, it contributes to overall sustainability efforts by minimizing food waste and resource utilization.

This study investigates the integration of non-destructive techniques, machine learning (ML) regression models, and packaging methods to assess and predict the shelf life of fresh date fruits. While previous studies have investigated shelf life estimation using various methods, this research focuses explicitly on date fruits. It introduces low-cost VisNIR spectral sensors for non-destructive assessment of internal quality attributes. Additionally, ML regression models are developed to predict the stages of fruit development and estimate shelf life based on observed quality attribute data. Furthermore, the study explores the effects of different packaging conditions, including modified atmosphere packaging and vacuum packaging. By combining these elements, the research provides a comprehensive approach that goes beyond previous efforts in the literature and offers new insights into accurate shelf life estimation and preservation techniques for fresh date fruits.

The main contribution of the current study lies in applying non-destructive techniques, machine learning (ML) regression models, and modified atmosphere packaging (MAP) to estimate and predict the shelf life of fresh date fruits. In addition, the study demonstrates the effectiveness of low-cost VisNIR spectral sensors for the non-destructive assessment of internal quality attributes, enabling continuous monitoring of fruit quality without sample destruction. The development of ML regression models enhances the accuracy of shelf life estimation by utilizing observed quality attribute data. Additionally, the research highlights the benefits of MAP in extending the shelf life of date fruits. These contributions provide valuable insights into optimizing fruit quality management and decision-making for optimal freshness and nutritional benefits of fresh date fruits throughout the supply chain.

TinyML delivers intelligence to low-memory and low-power tiny devices by enabling machine learning. This research proposes a new lightweight model for fresh date fruit shelf life estimation based on a low-cost, handy VIS/NIR range spectral sensor and CNN architecture deployable on any Microcontroller supported by the Edge Impulse Platform. The proposed model is trained and validated using the Edge Impulse cloud platform using an in-house dataset.

The main objectives of the current study are the following:

1. Assess the physicochemical attributes of date fruits throughout their storage period in various modified atmospheres and determine the shelf life for each storage condition.
2. Develop a low-cost, fast inference, and portable shelf life estimator using a TinyML-assisted 18-channel spectrometer.
3. Develop real-time predictive regression models trained from Edge Impulse utilizing the reflectance property to predict the shelf life of fresh dates.
4. Validate the results obtained using the developed predictive models against the observed laboratory results.

The rest of the paper is structured as follows: First, we introduce the Materials and Methods used in Section 2; Section 3 details the results and discussion. Section 4 concludes the work and Section 5 suggests future work.

## 2. Materials and Methods

### 2.1. Sample Collection and Preparation

The date samples at the Khalal stage (Khalas cv.) were harvested from the Date Palm Research Center of Excellence experimental farm, King Faisal University, Saudi Arabia (25.26809 N, 49.70847 E).

The harvested fruit samples were cleaned, sorted, and washed immediately after harvesting. Afterward, the samples were precooled at 24 °C in cold storage to be randomly moved to storage treatments. Once the selected fruits were precooled, the samples of approximately 250 g were placed into vacuum-sealed bags (150 × 200 mm) and in trays with a single layer of modified atmosphere packaging (MAP) (35 × 135 × 185 mm). The vacuum sealer (SH-6691, Swiss Home) was used to vacuum and seal the vacuum sealer bags after packing with fresh date samples. The trays were obtained from VC999 MAP Systems, CH–9100, Melonenstrasse 2, Herisau, Switzerland. The sealing machine model VC999 TS300, Bernhard Inauen, Herisau, Switzerland, was used to process the MAP trays by adding the gas mixtures to the samples and then sealing the MAP trays. The fresh date samples to be packaged were put manually in the trays. The MAP trays with the fresh date samples were evacuated, filled with modified gas before sealing, and sealed with non-permeable film (320 mm PA⁄PP 65 my).

The MAP treatments were unsealed trays (Control), vacuum-sealed bags (VSB), MAP with 20% CO_2_ and N balance (MAP1), and MAP with 10% O_2_, 20% CO_2_, and N balance (MAP2). The modified atmospheric concentrations of the gases were supplied from premixed gas cylinders. All treatments were stored in the cold storage room at 5 °C and room temperature (24 °C) for quality assessment. The study focused specifically on predicting shelf life for fresh dates based on the data from every week in the early stage of maturity within the suitable storage period for each storage temperature and method. For example, measurements are taken every week when dates have a longer shelf life. In the later stages, when the dates were closer to expiration, measures were taken more frequently, over shorter intervals, to capture any rapid changes in the attributes. Three replicates from each packing treatment were randomly evaluated before and after each storage period.

### 2.2. Physicochemical Attributes Measurements

The physicochemical attributes (moisture content, water activity, total soluble solids, total sugar, pH, and tannin) of the date fruit were evaluated before and after each storage period. These properties were analyzed in the Date Palm Research Center of Excellence fruit quality laboratories at King Faisal University, Saudi Arabia. The fruit moisture content (MC) was measured using a vacuum-drying oven (LVO-2041P, Daihan Labtech Co., Ltd., Namyangju-si, Gyeonggi-do, Republic of Korea) by drying a sample of 50 g of fruits under vacuum at 70 °C. The fruit sample weight was determined after 48 h to calculate the date fruit MC according to the Association of Official Analytical Chemists (AOAC) [34]. The total soluble solids (TSS) of the date fruits were measured using a digital laboratory refractometer (RFM 840, Richmond Scientific Ltd., Unit 9, Lancashire, UK) [33]. The fruit’s total sugar (TS) was measured using the anthrone–sulfuric acid colorimetry method. The absorbance was determined at 630 nm wavelength using a spectrophotometer (Genesys 20, Thermo Scientific, Waltham, MA, USA). The amount of TS in the sample was quantified using a standard graph constructed by plotting standard concentration on the *x*-axis vs. absorbance on the *y*-axis [35]. The pH of the fruit was measured using a pH meter (S400, Mettler-Toledo LLC, Columbus, OH, USA). The TC of the fruits was measured using a spectrophotometer (Genesys 20, Thermo Scientific, Waltham, MA, USA) at 750 nm wavelength based on the method described in [36]. The TC was determined by establishing a calibration curve by measuring absorbance at different known gallic acid concentrations [35].

### 2.3. Characteristics of Low-Cost Multiband Sensor

This section describes the features of an 18-channel multi-spectral sensor and the mapping of wavelengths to significant fruit attributes, which in turn were used to predict shelf life. The AS7265x chipset from AMS OSRAM, Austria, used for this experiment consists of an 18-channel Visible (VIS) to Short Wave Near Infra-Red (SWNIR) multi-spectral sensor used for detecting the physicochemical properties of fresh dates. This chipset has onboard optical filters whose spectral response is defined by on-device Gaussian Band Pass filters with 20 nm Full Width at Half Maximum (FWHM) value and with three optical sensors and 6 wavelengths for each optical sensor, totaling 18 wavelengths. The device wavelengths for each optical sensor are listed in Table 1. The sensors also have integrated programmable constant current-led drivers, through which light intensity can vary. The reflected light from the target is used to find the internal characteristics of the fruit.

**Figure 1:**
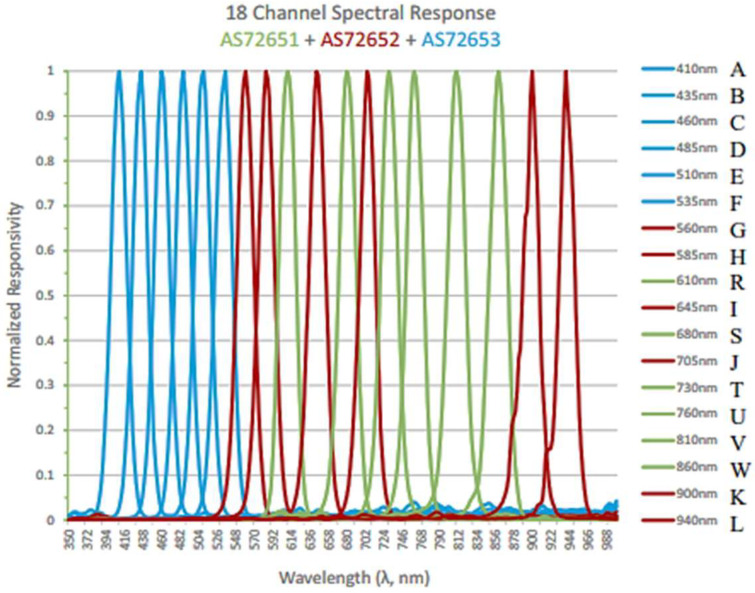
The responsivity of AS7265x—18-channel optical sensor [37].

The normalized responsivity for the entire spectrum of the Triad AS7265x chipset is reproduced from the manufacturer’s Datasheet in Figure 1. The normalized responsivity peaking occurs for various wavelengths and is coded with Alphabets “A, B---K, and L” [37].

**Figure 2:**
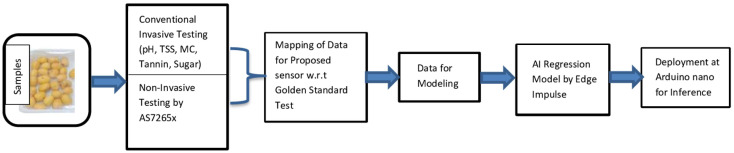
Methodology flow for the proposed shelf life estimation.

The wavelengths used in the AS7265x optical sensors correspond to a particular absorption peak of particular interest in this fresh date fruit maturity analysis (Khalal to Tamr). As reported by Giovenzana et al. [10] and Beghi et al. [11], 630 and 690 nanometers are near the characteristic peak of chlorophyll, 730 nanometers are near the third overtone of the -OH bond, and lastly, 810 and 860 nanometers are near the combination band of the -OH groups of sugars. Major attributes determining the shelf life of dates are pH, total soluble solids (TSS), sugar, MC, water activity (AW), tannin, and firmness. Water activity and moisture content in the fruit maturity stages were analyzed in different modified atmospheric conditions [36] and verified that the ratio (MC/aw) is 0.33. The non-invasive assessment of fruit firmness remains a “holy grail” in postharvest research [13]. According to Walsh et al. [13], a change in firmness is associated with minor changes in chemical composition, such as pectin levels. It is unlikely that NIRS can be used to detect these chemical changes in intact fruit. Thus, there is no consensus that firmness can be robustly (and directly) assessed using Vis-SWNIR. Finally, the major attributes considered for predicting the shelf life and analyzing the freshness of date fruits are pH, TSS, sugar, moisture, and tannin. Figure 2 shows the methodology flow for the proposed shelf life estimation. The date fruit samples at various modified atmospheres are subjected to both conventional testing for major attributes (pH, TSS, tannin, moisture content, and sugar) and the AS7265x sensors. The mapped data are used for AI model development through Edge Impulse flow. The final model is deployed on the Arduino Nano sense microcontroller for inferencing shelf life estimation.

### 2.4. Need for ML Models in Enhancing Food Sustainability

Broadly, the agriculture tasks are categorized into preharvest, harvest, and postharvest [38].

Machine learning evolved as a subdomain of Artificial Intelligence (AI) that comprises algorithms capable of deriving niche information from data and utilizing the same in self-learning to make good predictions or classifications. Machine learning and Artificial Intelligence can improve food sustainability by optimizing agricultural practices, such as precision farming, to reduce the resources used while maximizing yields. AI can also monitor crop growth and make more accurate predictions about yield, allowing farmers to plan better and reduce waste. Additionally, AI can be used in the supply chain to track food quality and reduce food waste by predicting customer demand and optimizing logistics for more efficient delivery.

The prime activities in each agriculture task and AI models used are listed below in Table 2.

The shelf life of food products mainly depends on environmental factors in the food supply chain. Environmental factors play an essential role in the dynamic change in the quality pattern of food products over time. Hence, the potential of incremental learning or lifelong machine learning approach may be utilized for building models with high classification or prediction accuracy. Lifelong learning (LL) involves a reinforcement learning approach and using the accumulated knowledge over time for future learning and solving problems [56].

To apply any machine learning approach, datasets, training, and Inference are the three pillars to improving food sustainability and reducing food waste.

**Figure 3:**
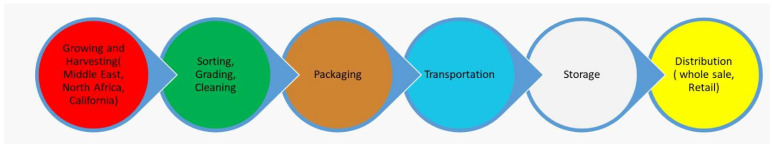
The supply chain of fresh dates.

Figure 3 shows the process of bringing food (fresh dates) from farmers to consumers. Throughout the process, various stakeholders, such as farmers, packaging companies, logistics providers, and retailers, work together to ensure the dates are safely transported and delivered to consumers. Similarly, various Internet of Things Sensors, actuators, and ML models are used to preserve the freshness of date fruit. However, the choice of Model development and deployment is decided by two approaches: Cloud computing and Edge computing.

### 2.5. Computing Choices for ML Model

**Cloud computing** is a model for delivering computing services over the internet. It allows users to access virtual resources such as computing power, storage, software, and services on demand without managing their own physical hardware.

**Edge computing** is an emerging computing paradigm that refers to a range of networks and devices at or near the user. Edge computing is about processing data closer to where it is being generated, enabling processing at greater speeds and volumes, leading to greater real-time action-led results.

The following parameters have the same choice of computing as in Table 3: We define the **“ITSBLERP**” parameters.

### 2.6. Need for Tiny Machine Learning

TinyML brings machine learning to microcontrollers and Internet of Things (IoT) devices to perform on-device analytics by leveraging the massive amounts of data they collect.

Tiny Machine Learning (TinyML), a rapidly growing subfield of applied ML, is a prime candidate for enabling computation and inference on edge devices. This budding area focuses on deploying simple yet powerful models on extremely low-power, low-cost microcontrollers at the network edge. TinyML models require relatively small amounts of data, and their training can employ simple procedures. Furthermore, as TinyML can run on microcontroller development boards with extensive hardware abstraction, such as Arduino products, deploying an application onto hardware is easy. TinyML enables diverse always-on applications ideal for battery-powered devices, particularly in Food supply chain verticals. Additionally, the cost-effectiveness and efficiency advantages of TinyML make it possible to deploy distributed TinyML systems that collaborate at the “edge” of the cloud computing network, making it a perfect fit for our proposed work [30,31].

### 2.7. How to Implement TinyML?

There are a couple of machine learning frameworks that support TinyML applications, as follows:

1. TensorFlow Lite for mobile-based applications
2. PyTorch Mobile
3. Tensor Flow Lite for Microcontrollers (TFLM)

Building ML models for mobile devices (using TensorFlow Lite) or the web (using TensorFlow.js) is possible using high-level programming languages such as Python and JavaScript. These languages are easy to learn and far more accessible to beginners than C/C++, which are microcontroller-friendly. However, the cost of mobile devices is higher than that of microcontroller devices. Also, optimizing and compressing Neural networks on mobile devices is not straightforward. On the other hand, popular frameworks such as TensorFlow Lite for Microcontrollers (TFLM) [30] help address optimization and compression of neural networks for embedded devices, but adoption has been slow due to challenges (data collection, data processing, development, deployment, and monitoring) that are unique to the embedded machine learning ecosystem. Edge Impulse, an online platform designed to simplify the process of collecting data, training deep learning models, and deploying them to embedded and edge computing devices, allows us to address these issues.

Due to the above-mentioned fact, we plan to develop our prediction models for shelf life estimation using Tiny Machine Learning supported by Edge Impulse Cloud MLOPs.

### 2.8. TinyML Development Using Spectral Sensor and Edge Impulse Platform

This research work proposes a low-cost portable exploiting TinyML models deployable on Arduino Nano 33 BLE Sense microcontroller for real-time prediction of shelf life in fresh date fruits, also known as “SSLED”. Visible- Short Wave Near Infrared range Spectrometric TinyML model; “SSLED”—Spectral Shelf Life Estimator for Dates. The developed models are targeted at the Arduino Nano33 BLEsense–Cortex M4 microcontroller that can run neural network models using TensorFlow Lite for microcontrollers (TFLM). Nano33 BLE sense board hosts numerous sensors (microphone, temperature, humidity, pressure, vibration, orientation, color, brightness, proximity, gesture, etc.) that enable a wide range of TinyML applications.

**Figure 4:**
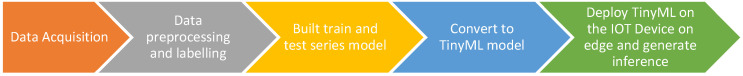
TinyML model workflow.

The end-to-end model development and deployment were carried out with the help of the Edge Impulse platform. The self-explanatory five stages used in TinyML model development are represented by flow diagram as in Figure 4.

### 2.9. Architecture of SSLED

**Figure 5:**
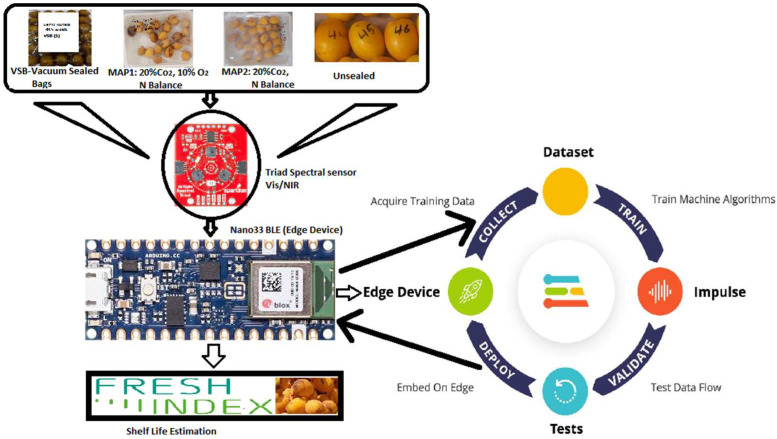
Block diagram of spectral shelf life estimator for dates (SSLED).

The schematic diagram displaying the complete architecture is presented in Figure 5. The 18-band spectral vis-SWNIR sensor captures the features (attributes) in reflectance values, indicating the amount of light reflected from the collected samples. These samples are transmitted to the Edge Impulse cloud platform through Arduino CLI on the Nano 33 BLE Sense (Edge Device).

The Edge Impulse cloud training platform performs the necessary training and deploys the model to the Edge device. The Nano 33 BLE then conducts real-time inference to estimate the shelf life, also called the “Freshness Index”.

The reflectance ratio is calculated using the measurement of incident light and the reflected light values obtained from the AS7265x—18-channel optical sensor. To measure incident light, we utilize a calibration process where we expose the sensor to a known light source. This allows us to establish a baseline value for incident light. The calibration ensures that we have a consistent reference point for measuring the reflectance of the dates.

Regarding the measurement of reflected light, the AS7265x sensor provides photonic values rather than direct reflectance values. However, these photonic values are proportional to the light the dates reflect. The sensor detects and quantifies light intensity across different channels, or spectral bands. By analyzing the photonic values for each channel, we can determine the reflectance characteristics of the dates and calculate the reflectance ratios.

### 2.10. Structure of Neural Network Used for Spectral Shelf Life Estimator for Dates (SSLED)

In this study, Tiny Machine Learning-based ANNs have been used to predict the shelf life of fresh date fruit. The reflected light from the AS7265x chipset corresponding to major physicochemical properties, i.e., date fruits’ pH, TSS, sugar, tannin, and MC, were taken as input for the regression NN model.

**Figure 6:**
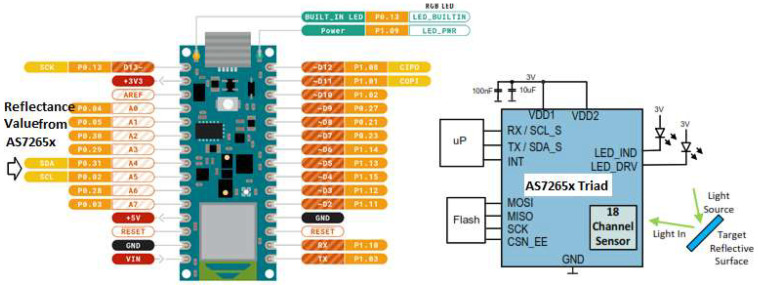
Connection schematic between spectral sensor and Arduino.

**Figure 7:**
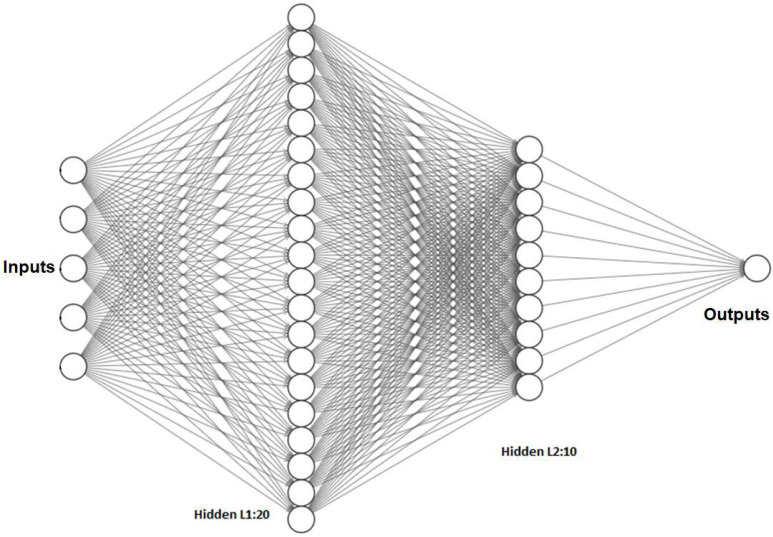
TinyML-based spectral shelf life estimator for dates (SSLED) neural network architecture.

The input layer acquires data from the AS7265x Triad optical sensor through the I2C port of the Arduino Nano33 BLE sense microcontroller (Edge Device). The connection diagram illustrating the spectral sensor to Arduino is shown in Figure 6. The neural network diagram of the TinyML SSLED prediction model is shown in Figure 7. The hidden layer performs the data transformation and feature extraction, and the output layer delivers the continuous predicted values and the shelf life/freshness index based on the major attributes of fresh date fruit.

This study utilized the Edge Impulse Cloud platform [37,38] to develop TinyML prediction models and assess their accuracy. Edge Impulse offers a straightforward approach to gathering data using built-in or external sensors in smart devices like mobile and embedded devices compared with other machine learning development platforms. It boasts a user-friendly interface, assists in data analysis, model design, and testing, and provides a deployable version of the model without requiring extensive coding knowledge, facilitating rapid prototype development.

The neural networks in the multilayer perceptron module were trained using a back-propagation learning algorithm with the Adam optimizer. The Adam optimization method adaptively estimates first- and second-order moments in stochastic gradient descent, efficiently updating the weights to minimize the error function.

The dataset was randomly divided into three subsets to train and evaluate the models: 60% for training, 20% for testing, and 20% for the holdout subset. The training dataset was used to determine the weights and build the model, while the testing data helped identify errors and prevent overtraining during the training process. Finally, the holdout data validated the artificial neural network prediction model.

### 2.11. Model Evaluation

In this work, we used root mean square error and mean absolute percentage error to evaluate the TinyML prediction model using the following equation:

(1) $$\mathrm{RMSE}=\sqrt{\frac{\sum_{i=1}^{n}{\left(O_{i}-P_{i}\right)}^{2}}{n}}$$

(2) $$MAPE=100\times \frac{1}{n}\times \sum_{i=1}^{n}\left|\frac{O_{i}-P_{i}}{O_{i}}\right|$$

where RMSE is the relative error, MAPE is the mean absolute percentage error, O_i_ is the measured value, n is the number of the measured values, and P_i_ is the predicted value of the target parameter data, i.

## 3. Results and Discussion

The importance of this study is to estimate the shelf life of fresh dates using low-cost, real-time machine learning-assisted sensing techniques.

The samples (Khalas cv.) collected were tested for major attributes with Laboratory standard tests, and the low-cost AS7265x Triad—18-channel spectral optical sensor was calibrated using the benchmark results. The AS7265x chipset was calibrated with diffused light and a pre-stored Hexadecimal string stored in EPROM given by the vendor before data acquisition.

### 3.1. Major Attributes for Shelf Life

The mean values of the major attributes of dates during the fruit ripening stage were tested in the DPRC laboratory, and their values are given in Table 4. These values were used as a guide to label the dataset. In the current study, we focused on the three main stages of fruit maturity to capture a representative range of date fruit properties spanning from the Khalal to the Tamr stage. These stages were carefully selected to include dates at various ripeness levels, allowing us to observe variations in fresh fruit shelf life estimations. We considered that the shelf life expires after the fresh fruits turn into the second maturity stage (Rutab) or the fruits are spoiled. To determine the shelf life intervals for the fresh or Khalal stage, we conducted measurements throughout the entire storage of the dates. The measurements were taken at regular intervals, weekly for specific periods, depending on the change in the significant attributes we monitored under each storage temperature and method. By incorporating measurements throughout the entire ripening stage period, we aimed to comprehensively understand the attribute variations and their correlations with the shelf life of fresh dates. This information allowed us to accurately label the values of significant attributes based on the specific shelf life intervals for the fresh fruits.

### 3.2. Major Attributes

**Figure 8:**
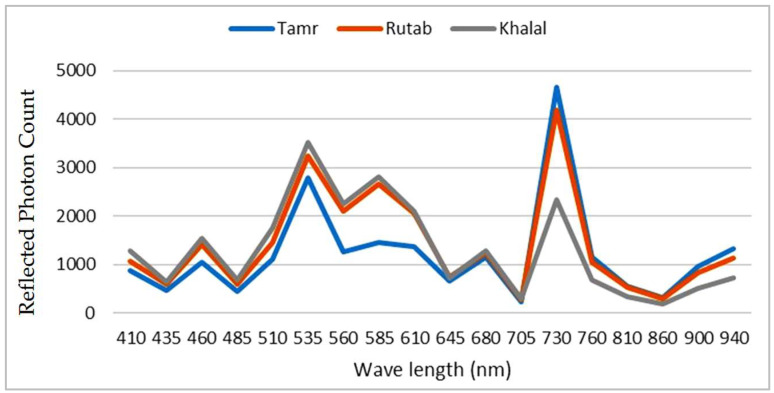
Reflected photon count versus wavelengths for three maturation stages of fruit.

The reflectance value (photon count) from samples read by 18 channels of optical sensors was recorded with an optical current source drive of 12.5 mA and a receiver gain of 64×. The 18-channel optical filters were swept at 20 nm incrementally from 410 nm up to 940 nm for each setting mentioned above (current and gain). Figure 8 presents the reflectance values from 18 spectral channels for three different stages of fruit maturity. The reflected photon count of the spectral sensor is reported on the *y*-axis for different wavelength settings of the sensor. All samples were tested under the same environmental light conditions.

From the laboratory results and reported results from related works [10,11,12,13,36,57], we found the following spectral bands sensitive to the significant attributes of date fruits, which are listed in Table 5. We termed the spectral band sensitive to each attribute as pH-SWNIR, TSS-SWNIR, Sugar-SWNIR, MC-SWNIR, and Tan-SWNIR.

The spectral measurements were conducted on the same day when the laboratory tests were conducted on the samples as they progressed through different maturity stages of date fruit, and the results matched the attributes of date fruits. The results were very convincing, and they prove the robustness of the Triad Spectrometer used for experimentation. The three-spectral sensor used is the AS7265x—triad sensor, which has three optical sensors (As72651, As72652, and AS72653), and hence we sum up all the three reflectance photon count values from the samples driven from White LED sources, NIR LEDs, and UV.

Having verified the correctness/matching of spectral bands with measurement results and identified the range of values for all major attributes during the fruit maturity stages, the dataset was used to train the neural network. At first, we verified the correctness or matching of the spectral bands. Spectral bands are specific ranges of wavelengths captured by the sensors used in the study. It is essential to validate that the spectral bands correspond accurately to the specific attributes or characteristics of the measured date fruit.

Next, we identified the range of values for all major attributes during various stages of fruit maturity. These attributes include moisture content, sugar level, pH, and other quality parameters relevant to the shelf life of date fruits. By determining the appropriate range of values for these attributes at different maturity stages, we established the target variables for the neural network to predict accurately.

Once the correctness and range of attribute values were confirmed, this dataset became the input for training the neural network. The neural network learns from this dataset by iteratively adjusting its internal parameters to minimize the difference between predicted and actual attribute values. Through this training process, the neural network becomes better at estimating the shelf life of date fruits based on the captured spectral information.

**Figure 9:**
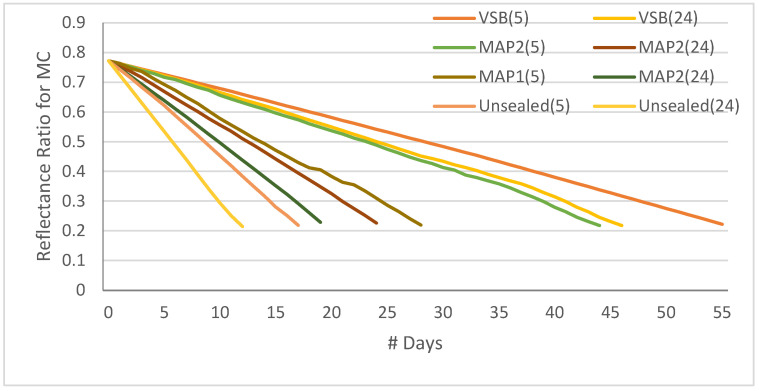
Reflectance ratio for moisture content for various treatments vs. shelf life in # days.

**Figure 10:**
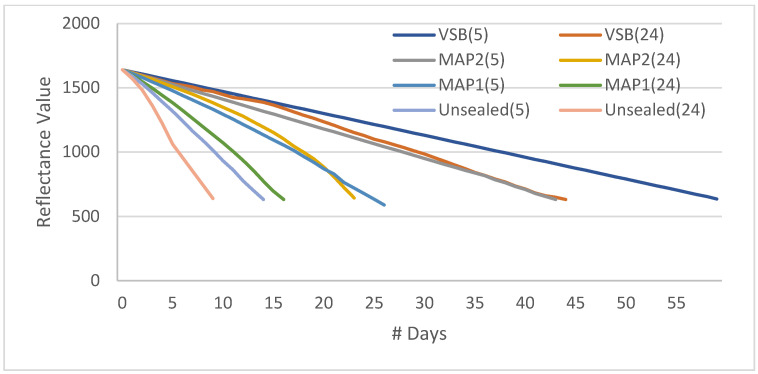
Cumulative reflectance value from the spectral sensor of five major attributes vs. shelf life in # days.

Even though we chose five attributes to estimate the shelf life of fruit, the moisture content is primarily responsible for fruit freshness/shelf life. So, we tried to plot the reflectance ratio of moisture content related to four treated samples maintained at 5 °C and 24 °C, totaling eight samples. Figure 9 shows the reflectance ratio for moisture content for various treatments. Reflectance ratio is the measure between incident light and reflected light; it gives the measure of absorption by sample under target. The Khalal stage (fresh fruit) reflected 80% of the incident light on the first day. When the fruit matures/ripens, the reflectance ratio deteriorates, indicating the degradation of freshness and, in turn, shelf life. The vacuum-sealed bags maintained at 5 °C showed the strongest resilience to fruit maturity/ripening, and it took 53 days to transform from khalal into the Tamr stage. In other words, we can say that the Glycemic Index of cv. Khalas was under control for 53 days. Whereas the unsealed sample kept at room temperature (24 °C) has lost its freshness in 10 days. From this graph, we can conclude that the cheapest and most affordable treatment is a vacuum-sealed bag, which is kept at room temperature and retains shelf life for 24 days. Figure 10 shows the plot for the cumulative reflectance value from the spectral sensor of five major attributes, and one can notice the same trend as in Figure 9 since moisture content is a major reason for fruit maturity and TSS and sugar content are influenced by moisture content.

### 3.3. Datasets for TinyML Model Development

The Edge Impulse platform provides a well-structured and simple step to building a model. It allows users to upload different types of preprocessed data. For this experiment on a predictive model, the raw data were uploaded and indicated as time series data. Since our data were used for regression problems, the data upload technique may differ from the familiar classification method. The dataset structure requires separate files (CSV), each named under its label, for all data points as integer values.

The reflectance value from spectral sensors of four categories of treated samples, respectively unsealed, vacuum sealed bags, MAP1, MAP2, and unsealed bags, was coded as a time series of data representing the significant attributes of fruit quality and labeled as a continuous value to represent the shelf life in the number of days as an integer value. The sample-labeled dataset structure coded as time series steps used for uploading the datasets to the Edge Impulse platform for untreated samples is shown in Table 6. Similarly, we labeled all categories of samples and uploaded them to the Edge Impulse cloud training platform. In all our discussions, if shelf life is 0, it is fresh fruit (Khalal—0th/starting day of ripening stage; 14th/nth day the fruit has fully ripened, that is matured to Tamr stage).

### 3.4. TinyML Model Development

The next step is model building. Regression models with the hyperparameters used for implementation are shown in Table 7. The model has to predict the shelf life of fruits, which are continuous values; hence, regression models were chosen. In addition, the edge device has memory limitations, and lightweight regression models were used.

Regression models are used for prediction, forecasting, and understanding the impact of independent variables on the dependent variable. They are widely used in various fields, including medical sciences, finance, social sciences, and machine learning. Here, regression models were utilized in shelf life estimation to understand the relationship between various factors and the deterioration of a product over time. In this work, we used continuous independent variables for regression models. These models help predict the remaining shelf life of a product based on factors such as temperature, humidity, packaging, and storage conditions. Here, the independent variables used are TSS, pH, water content, sugar, and tannin. The above features are extracted from the reflectance values of AS7265x sensors. The model parameters for the Neural Network block used to implement regression models are listed in Table 7.

The number of samples taken for VSB (5) (Vacuum Sealed Bag maintained at 5 °C) was 960 because the shelf life of the VSB samples is the longest (57 days for fruit ripening). Every week, we need samples for laboratory testing to validate the attributes of dates during the ripening stages. Similarly, untreated samples kept at room temperature have the least shelf life (10 days); 120 samples were sufficient for training. The batch size of 32 was chosen to reduce memory during the training and inference phases. The learning rate of 0.005 was chosen based on batch size and for better convergence. The ADAM optimizer is used because it has fast convergence.

The current study used the ReLU activation function, widely adopted in various ML applications, including neural networks. ReLU is an activation function used in neural networks, especially convolutional and deep neural networks. It is defined as y = max (0, x), which means it outputs the input value if it is positive and zero if it is negative. It is simple yet far superior to previous activation functions like sigmoid or tanh. ReLU has become the default activation function for many types of neural networks because a model that uses it is easier to train and often performs better. ReLU has shown superior performance in many scenarios and offers computational efficiency compared with other activation functions such as sigmoid or tanh. However, using adaptive activation functions is an interesting avenue to explore for enhancing the convergence and performance of neural networks. Adaptive activation functions adjust their parameters during the learning process and can adapt to the specific characteristics and complexity of the given dataset [58].

The Edge Impulse cloud platform is designed to deploy models for real-time applications on the edge device (Arduino Nano 33 BLE). Based on the hyperparameter used for the neural network model, the inference time is 1 milli second, and the RAM usage is 1.8k out of 256 KB to store model parameters. Also, the neural network model consumes only 10.9 KB out of the 1 MB of flash available. These numbers show that this model is optimized for TinyML implementation and for real-time inferencing.

The performance results, accuracy, and MSE for various confidence threshold settings for all treated categories of samples after training using all validation set samples are shown in Table 8.

The range of attribute values (moisture content, total soluble solids, and sugar) was almost 16 to 60 and was responsible for fruit maturity/ripening. However, the tannin value range changed between 0.3 and 6.19 and the pH value between 5.30 and 6.9, which were responsible for fruit maturity/ripening. Even though it looks linear, there is a small amount of nonlinearity during the lab test and a visible short wave near the infrared sensor. Hence, we introduced a nonlinear activating function (ReLu) in the neural network introduced in the tinyML flow. The accuracy plots for all modified atmosphere samples at different confidence levels were plotted with and without reluctant activations, and we conclude that ReLu is much needed for the SSLED sensor. To satisfy both the accuracy and lightweight model of the tiny sensor proposed, which is not a shallow network, we performed a sensitivity analysis for various hyperparameters (batch size, number of hidden layers, Adam optimizer, momentum), and from the results, we conclude that a bat with a size of 32, two hidden layers, and momentum of 0.9 offered better accuracy. The learning rate and epochs are not very sensitive. The learning rate was chosen as 0.005, and # epochs was 100 to converge.

**Figure 11:**
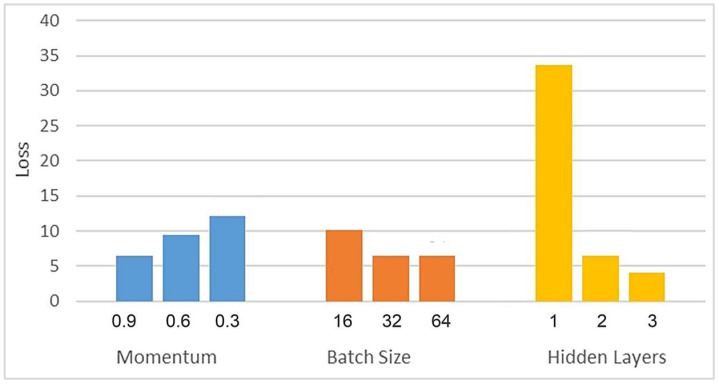
Sensitivity analysis for hyperparameters (momentum, batch size, and layer numbers).

Figure 11 presents the sensitivity analysis results for hyperparameters: momentum, batch size, and layer numbers. This analysis is crucial to understanding these hyperparameters’ impact on the model’s performance. The sensitivity analysis provides valuable insights into how changes in these hyperparameters affect the overall performance and accuracy of the model. The study assesses their influence on the model’s predictive capabilities by systematically varying each hyperparameter while keeping others constant. The results in Figure 11 allow us to identify optimal values for each hyperparameter, which can significantly enhance the model’s performance. This information is vital for fine-tuning the model and achieving the best possible accuracy while considering resource constraints.

**Figure 12:**
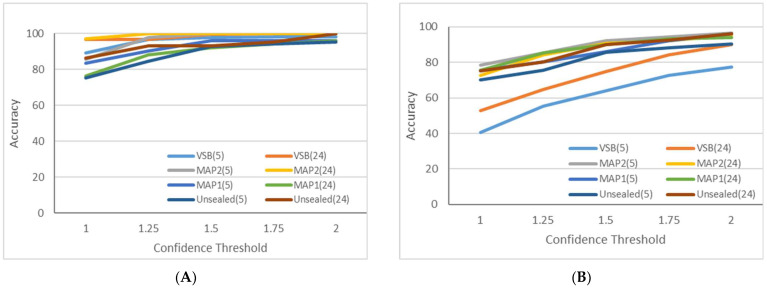
Model accuracy with activation function (**A**) and model accuracy without activation function (**B**).

Figure 12 compares the model accuracy with and without the activation function. Figure 12A shows that the activation function significantly benefits the model’s accuracy. The activation function introduces nonlinearity to the neural network, enabling the model to capture complex patterns and relationships within the data. As a result, the model’s accuracy improves, demonstrating the importance of selecting the appropriate activation functions for this task. Figure 12B shows the plot for the model accuracy without the activation function. Without an activation function, the model might struggle to learn complex patterns and perform poorly on the given task. This is because linear models can only learn linear relationships between features, limiting the model’s representation power. The results indicate that activation functions are crucial for higher accuracy and better generalization in the ANN models.

**Figure 13:**
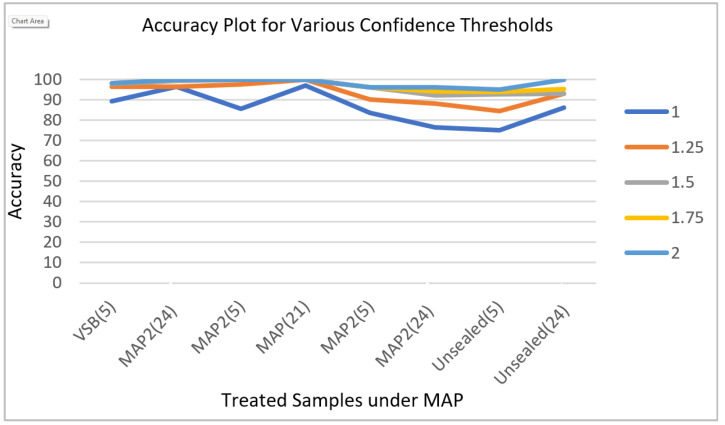
Model accuracy for treated samples for various confidence thresholds.

The confidence threshold setting suggested by Edge Impulse based on the number of samples in the dataset is 4.3, but we restricted it to lower levels. Increasing confidence threshold values will always yield better results. Since shelf life is reported in the number of days with a resolution of 1 day, the ideal confidence threshold should be 1. Out of all four treated samples maintained at 5 °C and 24 °C, MAP2(24) gives 97.13% accuracy with a MSE of 0.15. On the other hand, MAP1(24)-MAP trays sealed with 10% O_2_, 20% CO_2_, and N balance at room temperature yield 76.4% accuracy with a MSE of 0.68. The model accuracy plot is plotted for various confidence thresholds starting from ideal case 1 up to 2, with an increment of 0.25, as shown in Figure 13. This plot shows that even all the samples show more than 93% accuracy for a confidence threshold of 1.5, and even for an ideal threshold setting of 1, most of the sample’s model accuracy is greater than 85 percent, except for the MAP1(24) and unsealed (5) categories of samples. This proves that the model is robust. The reason for poor accuracy related to the MAP1(24) and unsealed (5) categories of samples could be that some of the samples could have been spoiled in that sealed bag due to bruises/friction during the cleaning process or some other internal attributes.

**Figure 14:**
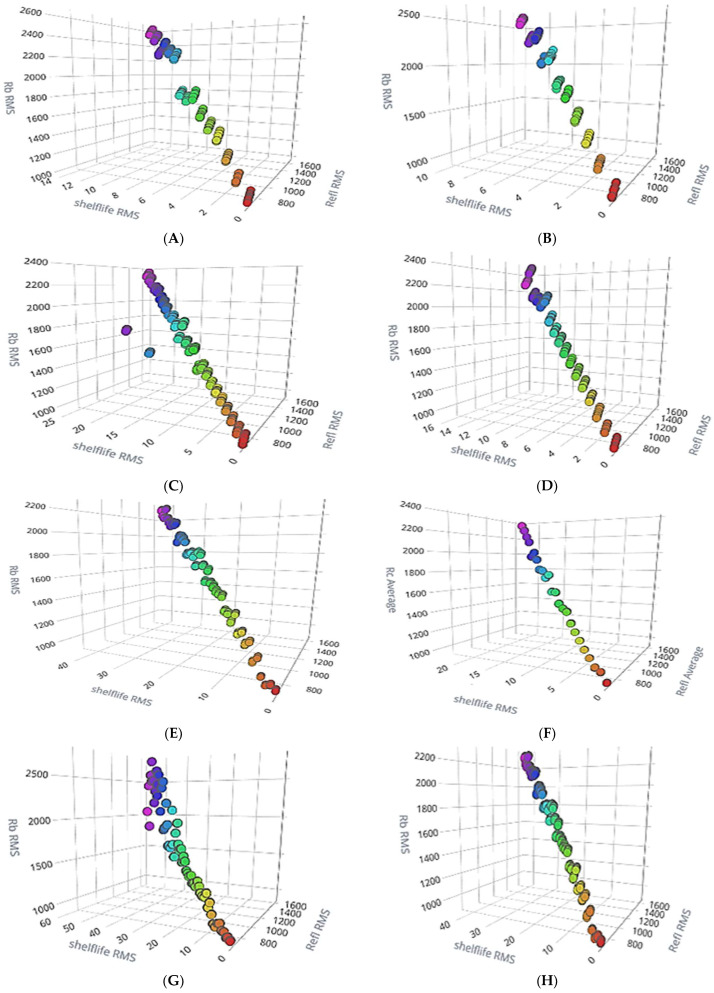
Data visualization plots covering four treated samples kept at cold storage room (5 °C) and at normal temperature (room temperature 24 °C). (**A**) unsealed at 5 °C, (**B**) unsealed at 24 °C, (**C**) MAP1 at 5 °C, (**D**) MAP1 at 24 °C, (**E**) MAP2 at 5 °C, (**F**) MAP2 at 24 °C, (**G**) VSB at 5 °C, and (**H**) VSB at 24 °C.

Using the feature explorer option of the Edge Impulse platform, the RMS value of reflectance concerning spectral sensors AS7261 and AS7262 and shelf life RMS are plotted for better data visualization for all eight samples and are given in Figure 14. From all those plots, we can see the linear relationships between the reflectance value of the spectral sensor and shelf life, confirming the robustness of the model. In all the following plots, the X-axis shows the shelf life over several days. From right to left, marked from 0–fresh fruit (Khalal)/starting day of ripening to last day/fully ripened (Tamr stage), the RMS value of reflectance (a.u) wrt AS7262 is marked as Rb RMS. Similarly, the RMS value of reflectance for AS7261 is marked as Refl RMS. The colors (blobs) given for the samples in Figure 14 were (VIBGYOR) and reported by the Edge Impulse studio application for better visualization. Violet for ripened fruit (more days) to Red for Khalal/fresh date fruit (0th day).

**Figure 15:**
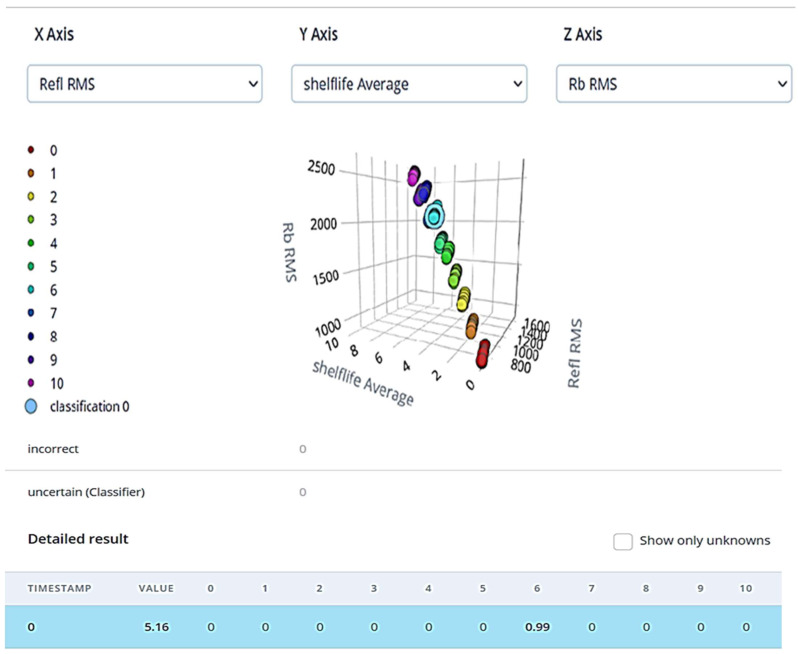
Live classification results for sample from unsealed tray kept at room temperature.

Figure 15 shows a snapshot of live classification results from Edge Impulse studio. The sample taken for the demonstration is from unsealed trays kept at 24 °C. The sample was labeled as having a shelf life of 6, predicted as 5.16, and is pretty accurate with a confidence of 0.99. The light blue color blob (bigger size) shows the live sample classification in the screenshot. The shelf life of samples kept in an unsealed tray at room temperature ranges from 1 to 10 days. Here, a shelf life of 0 days means the fruit matures fully (Tamr), whereas a shelf life of 10 days means it will take 10 days to reach the Tamr stage from the Khalal stage.

**Figure 16:**
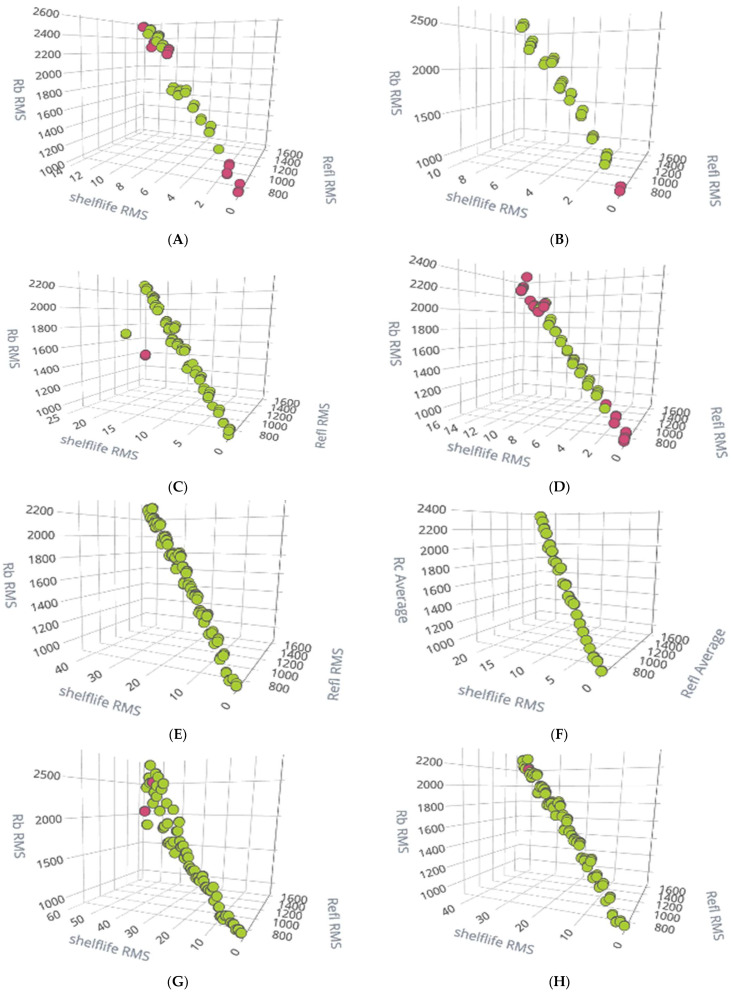
Model test results for all treated samples when confidence threshold was set to 1.5: (green blobs are correct; red ones are incorrect prediction). (**A**) unsealed at 5 °C, (**B**) unsealed at 24 °C, (**C**) MAP1 at 5 °C, (**D**) MAP1 at 24 °C, (**E**) MAP2 at 5 °C, (**F**) MAP2 at 24 °C, (**G**) VSB at 5 °C, and (**H**) VSB at 24 °C.

Figure 16 shows the eight subplots of model test results—data visualization covering four treated samples kept at a cold storage room (5 °C) and a normal temperature (24 °C). The green blobs are correctly predicted, and the red ones are wrongly predicted.

**Figure 17:**
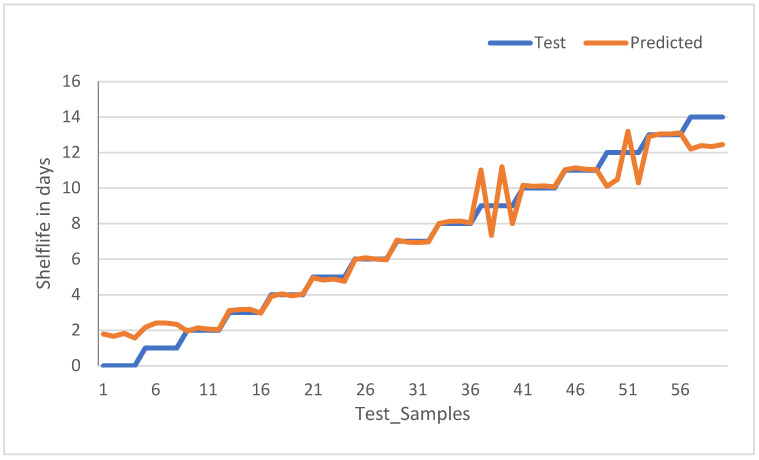
Model test results for unsealed samples at cold storage (5 °C).

**Figure 18:**
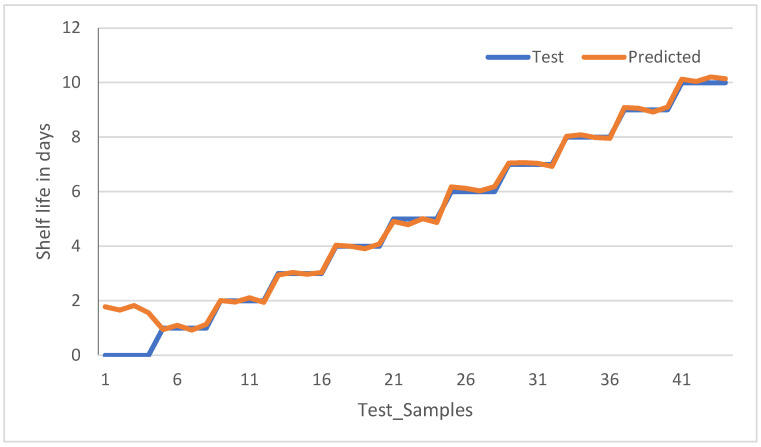
Model test results for unsealed samples at room temperature.

To have better insight in reading and interpreting the model test result performance, the results for all samples in unsealed (5 °C) and unsealed (24 °C) (the top two of the subplots of Figure 15) are plotted for shelf life in the number of days versus all the samples in that category and shown in Figure 17 and Figure 18, respectively.

Figure 17 shows the model test results of the unsealed sample kept at 5 °C. The observation is that samples 1 up to 4 are labeled (the test sample) as having a shelf life of 0, but the model predicted 1.78 days with an error of more than 100 percent. Similarly, sample numbers 36 to 39 are supposed to predict that a shelf life of 9 has an error of more than 100 percent. For samples 5–9 and 57–59, the error is less than 100 percent. The remaining other samples are predicted correctly, and these results are obtained with a confidence threshold of 1.5.

Figure 18 shows the model test results of the unsealed samples kept at 24 °C. The observation is that samples 1 up to 4 are labeled as having a shelf life of 0, but the model predicted 1.78 days, which has an error of more than 100 percent. The remaining samples are predicted correctly, and these results are obtained with a confidence threshold of 1.5.

From the subplots of Figure 16, we validate that the model performs adequately for most of the sample in four categories: VSB, MAP2, MAP1, and unsealed. There were few wrong predictions highlighted in Figure 17 and Figure 18; other than that, most samples were predicted correctly. We believe the wrong prediction is due to some other internal attribute of date fruit.

**Figure 19:**
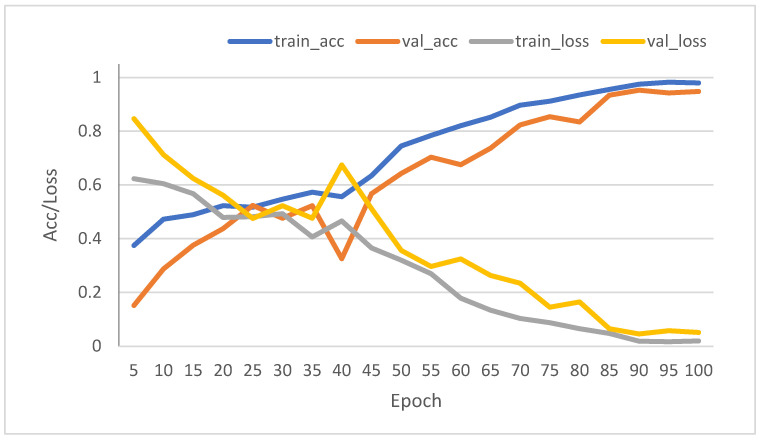
Training and validation accuracy and loss curves for VSB samples at room temperature.

The plot showing the accuracy and loss curve for both training and validation for 100 epochs during the training cycle is shown in Figure 19. After 90 epochs, the model converges very well and is the correct fit. This plot is generated with a seed of 31 (the initial weights and bias during training are random and vary with different seeds) during the training stage to report the model accuracy consistently.

We validated our model with lab results from the DPRC laboratory when we did live testing. To the best of our knowledge, we did not find any papers working on shelf life prediction using the TinyML regression model for shelf life prediction of fresh dates. However, several related studies exist for Royal Galas, strawberries, mangos, bananas, musk melons, and grapes using Computer Vision models and other classification models. Most of the work has reported around 95% accuracy. Our model results are also good, ranging from 95% to 100% for different confidence threshold settings. The selected microcontroller, Nano 33 BLE, was tested on a Cr2032 Lithium ion 3v3 battery (225 mAH) and consumes much less inferencing power. The TinyML kit will consume only 1–2 mA; however, during the sampling time, the spectral sensor LED is taking 12.5 mA. The battery will last for a week without recharging, assuming 10 min of sampling/inferencing per day. The data acquisition time is just 3 s with calibration, the inference time is less than 100 ms, and with a 225 mAH battery, it will last for a minimum of a week.

The challenge we faced in the TinyML training phase using the Edge Impulse platform was only uploading data for training models. Edge Impulse supports only the onboard sensors of selected microcontrollers/edge devices to acquire the data directly (live upload). The AS7265X sensor we used was an off-the-shelf sensor, and hence we had to acquire the data and store it on an SD card, then convert the reflectance value corresponding to all the attributes for shelf life prediction as a comma separated value (csv), which took a considerable amount of time in weekly sampling and testing. However, the model deployment was easy due to the Edge Impulse platform.

## 4. Conclusions

We conclude that simple vacuum packaging and low-cost shelf prediction—lightweight models with a low-cost spectral sensor (40 USD)—support edge computing, a key enabler to predict shelf life, thus ensuring sustainable nutrient food availability throughout the year. Consumers in food and agro-industry verticals such as quality management, production, storage, logistics, supply chain, and processing can choose the type of treatment and ML models (cloud/edge). A low-cost handheld spectral sensor integrated with an Arduino Nano 33 BLE microcontroller able to predict the shelf life of fresh fruit at all stages of the fruit ripening process has been experimentally verified for its robustness by validating against lab results. The accuracy of the lightweight regression model for all the treated samples was above 93% for a confidence threshold of 1.5. The reported performance shows that this TinyML sensor can be used as a handheld device for real-time prediction of the shelf life and freshness of the fruit.

## 5. Future Works

Ensemble Machine learning techniques that accommodate both vision and spectral-based sensors for shelf life estimation, which can cover all perishable food items in the supply chain and benefit a broad category of customers in the supply chain, may be attempted.

## Figures and Tables

**Table 1:** Multiband spectral sensor wavelengths.

Sensors	Wavelengths
AS72653	410 435 460 485 510 535
AS72652	560 585 645 705 900 940
AS72651	610 680 730 760 810 860

**Table 2:** Agricultural activity and AI model chart.

Task	Activities	Models
Preharvest (Health of Crop)	Soil, seed quality, fertilizer/pesticide application, pruning, cultivar selection, genetic and environmental conditions, irrigation, crop load, weed detection, and disease detection.	Artificial Neural Network (ANN), Fuzzy logic, decision trees, Naïve Bayes, k-means clustering, support vector machines (SVM), random forest (RF), k-Nearest Neighbor (k-NN), and XGBoost, Ensemble technique [35,39,40,41,42,43,44,45,46].
Harvesting	Fruit/crop size, skin color, firmness, taste, quality, maturity stage, market window, fruit detection, and classification.	Convolutional neural network (CNN), Resnet, Mobilenet, Densenet, long-short-term memory (LSTM), Recurrent Neural Network (RNN), Alexnet, LeNet, Linear Discriminant Analysis (LDA), and Principal Component Analysis (PCA) [12,16,22,23,25,27,36,39,47,48,49]
Post Harvesting	Factors affecting the fruit shelf-life include temperature, humidity, moisture conditions, gasses used in fruit containers, usage of chemicals in postharvest and fruit handling processes to retain quality, and fruit grading as per quality.	Linear Regression (LR), RNN, LSTM. Reinforcement Learning Models [47,50,51,52,53,54,55].

**Table 3:** Computing Decision Parameters.

Parameters	Cloud AI Computing	Edge AI Computing
**I**nference time	--	++
**T**raining time	++	--
**S**calability	++	+
**B**andwidth	--	++
**L**atency	--	+++
**E**conomics	-	++
**R**eliability	-	++
**P**rivacy	---	+++

+/- represent the favorable or not-so-favorable choice. Most of the Food and Agriculture chain activities favor Edge AI computing except for training.

**Table 4:** Attributes of dates during ripening stages.

Maturity Stage of Date Fruit	The Mean Value of Major Attributes of Dates				
	pH	TSS (Brix)	Sugar (%)	MC (%)	Tannin (%)
Khalal	5.30	24.86	24.96	71.47	6.19
Rutab	6.15	51.29	52.02	46.54	1.05
Tamr	6.64	60.58	63.35	16.94	0.3

**Table 5:** Wavelength table for attributes of fresh fruit.

Major Attribute		Wavelength in nm
Number	Terminology/Name	
1	MC-SWNIR	535, 705, 940
2	pH-SWNIR	510, 680, 900
3	Sugar-SWNIR	460, 645, 810
4	Tan-SWNIR	560, 585, 610
5	TSS-SWNIR	410, 560, 730

**Table 6:** Labeled dataset for untreated sample (control) at 5 °C.

MC-SWNIR	pH-SWNIR	TSS-SWNIR	Sugar-SWNIR	Tan-SWNIR	Shelflife
1087	280	787	797	430	0
1065	282	797	807	417	1
1043	285	808	817	403	2
1021	287	819	827	388	3
999	289	829	837	376	4
977	292	840	847	360	5
955	294	850	857	349	6
933	297	861	867	333	7
911	299	872	877	322	8
889	302	882	887	305	9
867	304	893	897	293	10
845	307	903	907	277	11
823	309	914	917	259	12
801	312	924	927	243	13
779	314	935	937	231	14

**Table 7:** Model parameter for NN Block.

Parameters	Specifications							
Model Type	Sequential							
Input layer	15 major features + 3 (Vacuum, MAP2, MAP1)							
First level Hidden Dense layer	20 neurons							
Second level Hidden Dense Layer	10 neurons							
Dropout rate	0.2							
Output Layer	1 neuron (Y-Predicted, no activation function)							
Learning Rate	0.005							
Activation function for all layers	ReLu							
Batch Size	32							
Epochs	100							
Optimizer	Adam							
Loss function	MSE (Mean Squared Error)							
Number of Training Cycles	100							
Treatments	VSB (5)	VSB (24)	MAP2(5)	MAP2(24)	MAP1(5)	MAP1(2)	Unsealed (5)	Unsealed (24)
Training Dataset (80%)	960	706	706	448	416	272	240	120
Testing and Validation Dataset (20%)	240	178	178	112	104	68	60	30

**Table 8:** Evaluation of the prediction accuracy for the TinyML model.

Packing Type	Temperature	Threshold					
		Metrics	1	1.25	1.5	1.75	2
VSB	5	MAPE	89.39	96.6	97.87	98.3	98.3
		RMSE	0.39	0.39	0.39	0.39	0.39
	24	MAPE	96.65	96.65	99.44	100	100
		RMSE	0.23	0.23	0.23	0.23	0.23
MAP2	5	MAPE	85.8	97.73	100	100	100
		RMSE	0.39	0.39	0.39	0.39	0.39
	24	MAPE	97.13	100	100	100	100
		RMSE	0.15	0.15	0.15	0.15	0.15
MAP1	5	MAPE	83.65	90.38	96.15	96.15	96.15
		RMSE	0.61	0.61	0.61	0.61	0.61
	24	MAPE	76.4	88.2	92.18	94.16	96.12
		RMSE	0.68	0.68	0.68	0.68	0.68
Unsealed	5	MAPE	75.2	84.67	92.76	94.1	95.2
		RMSE	0.69	0.69	0.69	0.69	0.69
	24	MAPE	86.36	93.18	93.18	95.45	100
		RMSE	0.65	0.65	0.65	0.65	0.65

## Data Availability

The data are available upon request from the corresponding author.

## Nomenclature

A. U.	Arbitrary Unit
AI	Artificial Intelligence
ANN	Artificial Neural Network
CNN	Convolutional Neural Network
DT	Decision Trees
ET	Ensemble Technique
GI	Glycemic Index
IoT	Internet of Things
IR	Infrared Red
ITSBLERP	Inference, Training, Scalability, Bandwidth, Latency, Economics, Reliability, and Privacy Characteristics
K-MC	K-Means Clustering
K-NN	K-Nearest Neighbor
LDA	Linear Discriminant Analysis
LI	Lifelong Learning (Ll)
LR	Linear Regression
LSTM	Long Short-Term Memory
MAE	Mean Absolute Error
MAP	Modified Atmosphere Packaging
MC	Moisture Content
ML	Machine Learning
NB	Naïve Bayes
NIR	Near-Infrared Red
PCA	Principal Component Analysis
MAPE	Mean absolute percentage error
RF	Random Forest
RLM	Reinforcement Learning Models
RMSE	Root Mean Square Error
RNN	Recurrent Neural Network
SC	Sugar Content
SSLED	Spectral Shelf Life Estimator For Dates
SVM	Support Vector Machines
SWNIR	Short-Wave Near-Infrared
TC	Tannin Content
TFLM	Tensor Flow Lite for Microcontrollers
TinyML	Tiny Machin Learning
TSS	Total Soluble Solids
wa	Water Activity
Xgboost	Extreme Gradient Boosting
